# Delineation of Cancer Service Areas Anchored by Major Cancer Centers in the United States

**DOI:** 10.1158/2767-9764.CRC-22-0099

**Published:** 2022-05-24

**Authors:** Changzhen Wang, Fahui Wang, Tracy Onega

**Affiliations:** 1 Department of Geography & Anthropology, Louisiana State University, Baton Rouge, Louisiana.; 2 Department of Population Health Sciences, University of Utah, Salt Lake City, Utah.; 3 Huntsman Cancer Institute, Salt Lake City, Utah.

## Abstract

Defining a reliable geographic unit pertaining to cancer care is essential in its assessment, planning, and management. This study aims to delineate and characterize the cancer service areas (CSA) accounting for the presence of major cancer centers in the United States. We used the Medicare enrollment and claims from January 1, 2014 to September 30, 2015 to build a spatial network from patients with cancer to cancer care facilities that provided inpatient and outpatient care of cancer-directed surgery, chemotherapy, and radiation. After excluding those without clinical care or outside of the United States, we identified 94 NCI-designated and other academic cancer centers from the members of the Association of American Cancer Institutes. By explicitly incorporating existing specialized cancer referral centers, we refined the spatially constrained Leiden method that accounted for spatial adjacency and other constraints to delineate coherent CSAs within which the service volumes were maximal but minimal between them. The derived 110 CSAs had a high mean localization index (LI; 0.83) with a narrow variability (SD = 0.10). The variation of LI across the CSAs was positively associated with population, median household income, and area size, and negatively with travel time. Averagely, patients traveled less and were more likely to receive cancer care within the CSAs anchored by cancer centers than their counterparts without cancer centers. We concluded that CSAs are effective in capturing the local cancer care markets in the United States. They can be used as reliable units for studying cancer care and informing more evidence-based policy.

**Significance:**

Using the most refined network community detection method, we can delineate CSAs in a more robust, systematic, and empirical manner that incorporates existing specialized cancer referral centers. The CSAs can be used as a reliable unit for studying cancer care and informing more evidence-based policy in the United States. The cross-walk tabulation of ZIP code areas, CSAs, and related programs for CSAs delineation are disseminated for public access.

## Introduction

Cancer care assessment, planning, and management require a standardized system of geographic units on which reliable analyses can be conducted to address many challenges of the U.S. cancer care system (1). Since 2012, the NCI has mandated its designated cancer centers to identify and describe their catchment areas (CA) to address the cancer burden, risk factors, incidence, morbidity, mortality, and inequities within the CAs (2). In response, many centers either treated surrounding counties or an entire state as their CAs or combined the two (3). However, the rationale for defining the boundary was unknown and the self-declared CAs were not suitable for consistent comparisons at the national scale. Prior studies have concretized geographic unit for health care markets into various terms, such as hospital service areas (HSA; ref. 4), hospital referral regions (HRR; ref. 4), and primary care service areas (ref. 5). These units either lacked timely updates to meet the challenges of the ever-changing health care market or were not suitable for the highly specialized cancer care. More importantly, they were defined by the plurality rule that only emphasized the greatest interaction between patients and providers and lacked a systematic perspective of how to resolve uncertainties in the method to define them and standardize the delineation process (6). These concerns were addressed in recent studies (6–8) that developed novel data-driven and scale-flexible methods to define cancer service areas (CSA), within which cancer care utilizations among underlying populations were tightly tied to the service providers (7, 9, 10). A pilot study refined a recently developed effective network community detection method by accounting for spatial adjacency and other desirable constraints, termed “spatially constrained Leiden (ScLeiden) method,” and proved to be highly effective and efficient in delineating CSAs at various scales in the Northeast United States (7). However, the question remains as to what the appropriate number of CSAs is when extending the analysis to a national scope, and whether the presence of major cancer centers needs to be considered. This study addresses these two important aspects of delineating CSAs.

Cancer centers form the backbone of the cancer care system in the United States. The current 71 NCI-designated Cancer Centers (NCI-CC) in 36 states and the District of Columbia are dedicated to advancing cancer research and treatment and promoting community outreach and engagement. Their reputation for achieving better outcomes attracts more than 250,000 patients diagnosed or treated on average at an NCI-CC each year (around 16 million patients for all NCI-CCs that provide clinical care; ref. 11). The 71 NCI-CCs and 32 other cancer centers form the 103 members of the Association of American Cancer Institutes (AACI), and are premier academic and freestanding cancer centers which serve as major hubs of cancer research and care in the United States and Canada. These AACI-CCs are simply referred to as CCs hereafter. Prior studies found that the utilization of these centers was associated with proximity (12) and varied across regions and populations (13, 14). However, without defining their service areas, within which they draw most of their patients, reliable analysis of variabilities across their CSAs, as well as between the CSAs with and without any CC, is not feasible.

This study aimed to use the ScLeiden method, automated in a Geographic Information Systems (GIS) tool (6), to delineate a set of scientifically sound and reliable CSAs that best captured the local cancer care markets in the United States. The study differs from previous research as it made use of the scale flexibility of the ScLeiden method twice by accounting for the presence of the CCs. Special efforts were made to ensure that the CCs, banning close proximity, had their distinctive CSAs, and the remaining areas formed other CSAs with no anchoring CCs inside. The derived CSAs were assessed by indices such as population and area sizes, geometric shape, urbanicity, median income of residents, and average travel time, localization index (LI), and market share index (MSI) of service utilization.

## Materials and Methods

### Data Sources and Processing

The cancer care utilization data were extracted from the Medicare enrollment and claims from January 1, 2014 to September 30, 2015 provided by the Centers for Medicare & Medicaid Services (CMS) with an approved Data Use Agreement and Institutional Review Board protocol. The ending date was cut off because of the transition of new codes of International Classification of Disease (ICD; ref. 15). Medicare beneficiaries were ascertained from the Medicare beneficiary denominator file by identifying those who were ages 65–99 years old, continuously enrolled in fee-for-service, enrolled in both Medicare Parts A and B, and with no missing ZIP codes in 50 states and the District of Columbia. Then cancer care services (utilizations) that were defined as cancer-directed surgery, chemotherapy, and radiotherapy were identified from Medicare Provider Analysis and Review (MedPAR), outpatient, and carrier files using ICD-9-CM procedure (16), Current Procedure Terminology (CPT-4), and Healthcare Common Procedure Coding System codes. The cancer care providers that included hospitals, ambulatory surgical centers, and outpatient facilities were extracted from the Provider of Services files provided by CMS. By linking the Medicare beneficiaries as the origins and cancer care providers as the destinations to the Medicare claims of cancer care utilization, an initial spatial network was created from the locations of patients with cancer to the locations of cancer care providers with the total claims as the service volumes between them.

Considering ZIP code was the most granular feasible unit in Medicare data, the locations of cancer patients’ residences and cancer care providers were geocoded at the ZIP code level. Point ZIP codes (typically associated with large business entities) were aggregated to the ZIP code areas that enclosed those points. The ZIP code areas were extracted from the 2015 ZIP Code Tabulation Areas. Each ZIP code area had the point location represented by its population weighted centroid calibrated from the 2010 census block population data for achieving better accuracy in the rural or suburban areas with uneven populations (17). Thus, the initial spatial network can be represented by the ZIP code centroids of patients with cancer as origin nodes and ZIP code centroids of cancer care providers as the destination nodes, and the total service volumes (or claims) as the edge weights between each other.

However, the majority (70.15%) of flows had volumes < 11 and were suppressed based on the CMS data use agreement. To define reliable and meaningful CSAs, we interpolated these flows using the strategy proposed by Wang and colleagues (6, 9). We first estimated the travel time between ZIP code areas from a national drive time matrix that accounted for the hierarchical structure of road network and real-time traffic (18). We then used a spatial interaction model to derive the best-fitting distance decay function from the remaining flows (29.85%) with volumes ≥ 11 and interpolated the suppressed ones. As a result, our estimated flow accounted for 16.6% of the total service volumes in the spatial network. We also created a spatial adjacency matrix based on the contiguity of ZIP code areas that accounted for the availability of transport modes (6).

The cancer center data were downloaded and geocoded from the member directory of AACI, comprised of 103 leading cancer centers in North America (19). This study excluded 11 (two in Canada, one in Puerto Rico, and seven basic laboratories and the St. Jude Children's Research Hospital in the United States) and unfolded Louisiana Cancer Research Consortium of New Orleans at Stanley S. Scott Cancer Center and Tulane Cancer Center. A total of 94 CCs were identified.

### GIS Automated ScLeiden Method

In network science literature, there are many network community detection methods including hierarchical agglomeration (20), simulated annealing (21), Infomap (22), Louvain (23), Leiden algorithms (24), and their variants (7). Among them, the ScLeiden method has been demonstrated to outperform other well-known methods in defining high-quality service areas with high efficiency and effectiveness (6–8). Because of the recent advances in GIS, the ScLeiden method has been automated in a GIS tool, and used in this study to delineate CSAs in the United States (6).

In a brief, given the spatial network of cancer care utilization from the ZIP codes of patients with cancer to the ZIP codes of cancer care providers in our case, the ScLeiden method optimized modularity to group a set of densely connected and spatially contiguous ZIP codes in terms of the utilizations between into preliminary CSAs and continue to group densely connected preliminary CSAs into larger CSAs until no further improvement of modularity can be made and each CSA reached minimal region size. Thus, the service volumes (or utilizations) were maximal between each derived CSAs and minimal between CSAs. This study chose a threshold population of 120,000 as the minimal region size that was used to define HRRs because CSAs were similar but more specific to referral cancer care. The modularity, a quality measure to guide the process of delineating CSAs, was defined to capture the difference between the fraction of the total service volumes within CSAs and the fraction of the total service volumes between CSAs. Mathematically, it was formulat-ed as:



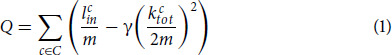



where *Q* represented the modularity value that summed over each CSA *c ∈ C, m* was the total number of service volumes in the spatial network, 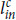 was the total number of service volumes between all ZIP codes within the CSA 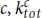 was the sum of the service volumes between ZIP codes in the CSA *c* and ZIP codes in other CSAs. The constant *γ* > 0 was the resolution parameter. One may increase its value to define a series of spatially contiguous CSAs at different scales, thus enabled the ScLeiden method being scale flexible.

### Delineating CSAs Using ScLeiden Method

The whole processing of delineating CSAs in this study contained three steps. We first used the scale flexibility of the ScLeiden method to delineate as many CSAs as the CCs. For each of the initial CSAs that contained multiple CCs, our second step was to extract flows within it to form a new subnetwork and then use the scale flexibility of the ScLeiden method again to further divide such a CSA into two or more sub-CSAs so that no two CCs were > 30 minutes apart and each sub-CSA had local utilization rate (LUR) ≥ 0.5. The 30-minute travel time was selected as a criterion following the prior studies (12, 25, 26) although it was open to debate. The travel time between CCs was estimated via Google Maps. Similar to LI, LUR referred to the proportion of service volumes within a sub-CSA out of total service volumes originated from the same sub-CSA and ended at any CSAs within the subnetwork. In other words, LUR was calculated for the sub-CSAs based on the service flow volumes within a subnetwork, thus it was a local indicator. LI was calculated for the CSAs based on all service volumes within the entire network, thus it was a global indicator. The 50% of LUR was used in other service area delineations (27). In our third step, we combined the initial CSAs without the need of further division from the first step and sub-CSAs from the second step to form the final set of CSAs, within which the service volumes were maximal but minimal between.

### Statistical Analysis

The main outcome measures were LI and MSI that were commonly used to measure the characteristics of health care service areas (28–30). LI was defined as the ratio of total service volumes within a CSA divided by the total service volumes originated from the same CSA and ended at any CSAs. As a population-based index, a higher LI was more favorable as it indicated a higher degree of utilization of local care, and a CSA therefore more effectively captured its cancer care market. MSI referred to the proportion of incoming service volumes from outside of a CSA over the total service volumes originated from any CSAs and ended at the same CSA. As a hospital-based index, a lower MSI implied that the hospitals in a CSA were less attractive to patients outside the CSA, and thus a more favorable CSA delineation. Prior studies also used net patient flow to capture the patient movement across regions (28–30). Because it can be inferred from LI and MSI, we omitted its discussion here.

A systematic literature review also suggested population, urbanicity, travel time, area, and income affected health behaviors and outcomes (5, 10, 29). We selected seven independent variables: population, population density, urbanization ratio, average travel time, area size, area compactness, and median household income to characterize the CSAs. Note that population, population density, and median household income were calculated on the basis of all age population. Average travel time was the weighted average travel time for patients with cancer originated from the CSA to any CSAs. Mathematically, it was defined as the sum of the actual travel time of patients with cancer multiplied by the associated service volume divided by the sum of service volumes originated from the same CSA to any CSAs.

To assess possible differences between CSAs anchored by CCs and those without CCs, we compiled descriptive statistics of all variables for the two groups, and compared their values using a *t* test. For all CSAs, the global Moran I value of LI or MSI indicated no significant spatial autocorrelation, and therefore a correlation analysis was used to examine the association between either of them and each of the independent variables, and a stepwise regression was further used to examine the collective effect of independent variables on LI or MSI. All analyses were performed in ArcGIS Pro and R software. All statistical tests were two tailed.

### Data Availability

The data generated in this study are publicly available via https://faculty.lsu.edu/fahui/news/2022/usa-110csa.php.

## Results

**FIGURE 1:**
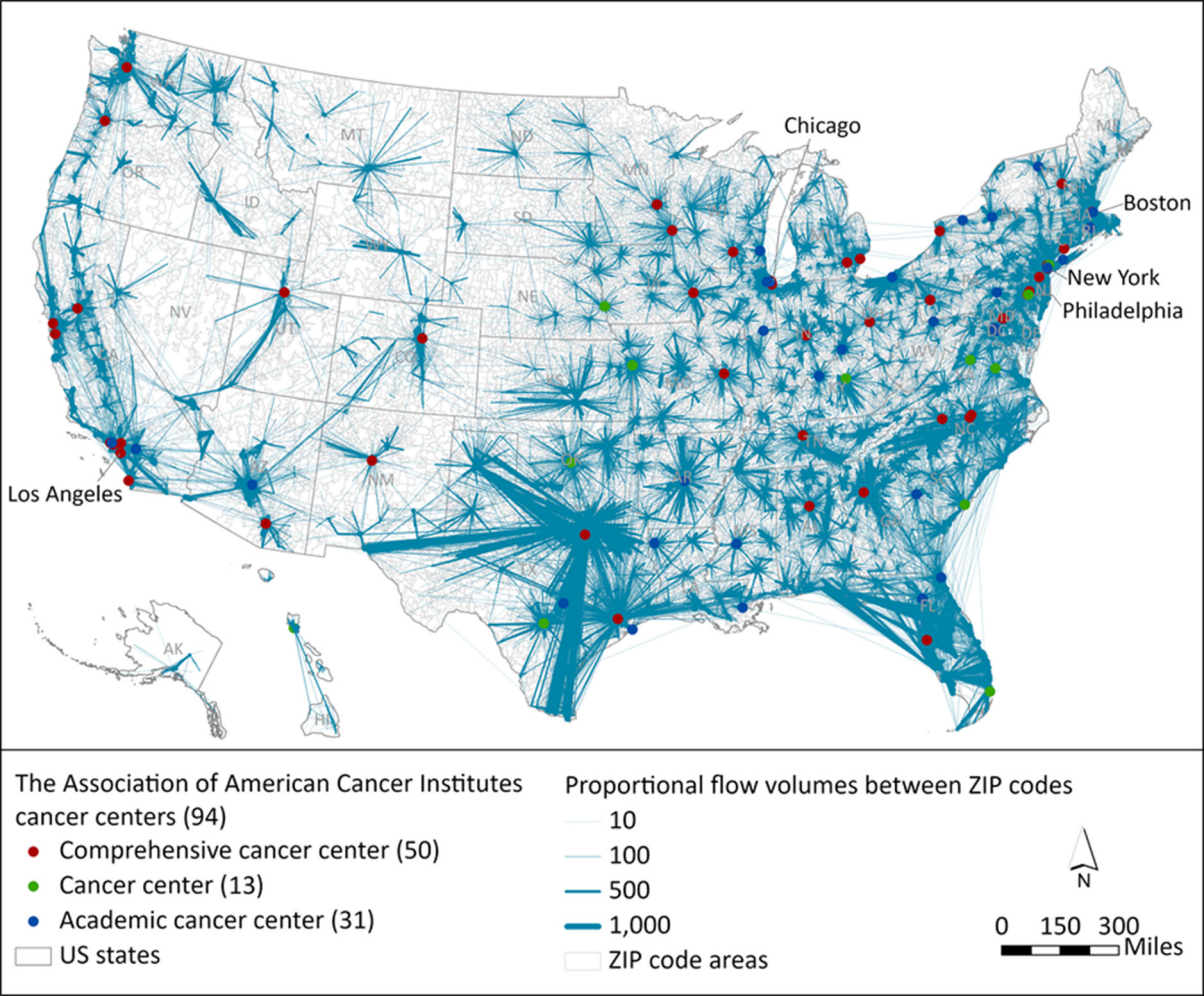
Spatial network of major cancer care service flows (volumes ≥ 30 and travel time ≤ 12 hours) overlaid with 94 CCs. Blue lines represented edges with widths proportional to service flow volumes between ZIP code areas of patients with cancer and ZIP code areas of cancer care providers at the two ends. Colored dots were locations of CCs.

The spatial network was composed of 32,989 nodes with total population of 308,774,408 and 520,960 edges (or flows) with a total service volume of 13,581,725. The 94 CCs included 50 NCI comprehensive cancer centers, 13 NCI cancer centers, and 31 academic cancer centers (Fig. 1). They were in 42 states with the most (*N* = 10) in California or New York, second most (*N* = 6) in Texas, and third most (*N* = 5) in Illinois or Pennsylvania. Nine states (Alaska, Nevada, Idaho, Montana, Wyoming, North Dakota, South Dakota, Delaware, and Maine) did not contain any CCs. Many hub-and-spoke subnetworks with large flow volumes were anchored by a stand-alone CC except those in Los Angeles, Chicago, Boston, New York, and Philadelphia that contained multiple CCs. Some subnetworks were intertwined in highly urbanized areas, such as those in northeast coast states, Florida, Texas, and southern California. Many service flows crossed state borders.

The ScLeiden method first delineated 94 CSAs as there were 94 CCs, among which 32 CSAs had no CCs, 45 CSAs contained one CC each, and 17 CSAs contained multiple (≥2) CCs each. The ScLeiden method was used again to delineate each of the 17 multi-CC CSAs into a series of sub-CSAs using resolution values ranging from 0.1 to 2 with an increment of 0.1 to assess whether it was feasible to derive a distinctive CSA for each CC. A higher resolution value corresponded to a larger number of sub-CSAs. In other words, each of the 17 CSAs, treated as a study area, was divided into a number of sub-CSAs that could not be further divided (i.e., CCs in each sub-CSA in close proximity, a threshold value 0.5 for LUR, and a minimum population of 120,000). As a result, eight CSAs stayed intact, and nine other CSAs were further divided. For the latter scenario, the nine initial CSAs were segmented into 25 sub-CSAs.

**FIGURE 2:**
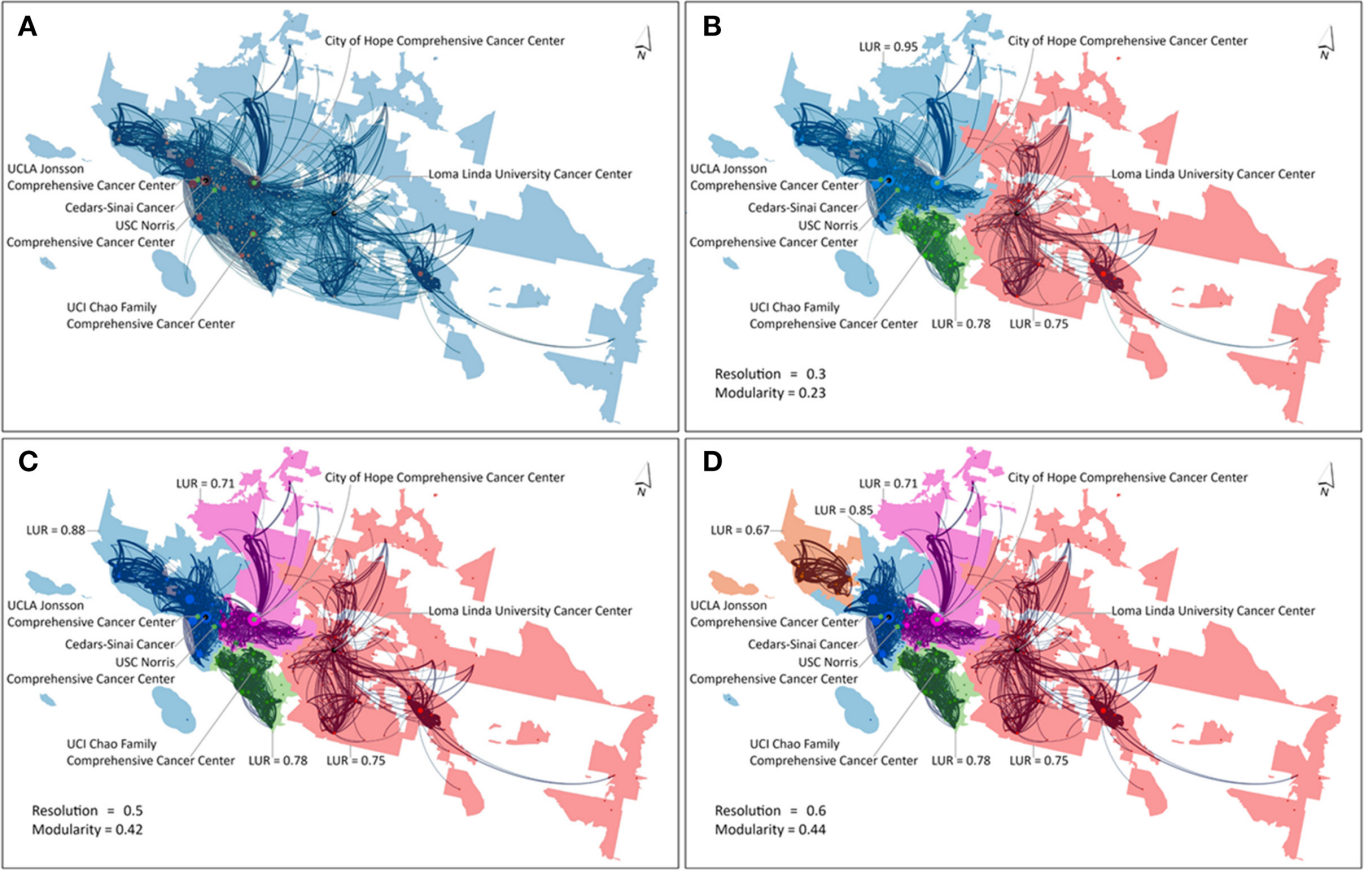
Delineating the CSA containing six CCs in Los Angeles into sub-CSAs. One initial CSA (blue color) in Los Angeles (**A**), three colored sub-CSAs with resolution = 0.3 or 0.4 (**B**), four colored sub-CSAs with resolution = 0.5 (**C**), and five colored sub-CSAs with resolution = 0.6 (**D**). Each CSA or sub-CSA was overlaid with service flow volumes ≥ 30 and six CCs. (Dot sizes were proportional to service volumes at CC hospitals, line width represented service volumes, and service flows between CSAs were negligible. The white areas represented large water bodies or large unpopulated land areas.)

Here the CSA containing six CCs in Los Angeles was selected to illustrate the process (Fig. 2A). No sub-CSAs were formed until resolution = 0.3 that yielded all three sub-CSAs (Fig. 2B): two sub-CSAs contained one CC each, and one large sub-CSA (in blue) had four CCs, some of which were > 30 minutes apart. When resolution = 0.5, 4 sub-CSAs were formed (Fig. 2C): the large sub-CSA from the previous round was split into two, such that no sub-CSA could be further divided (each with either one CC or multiple CCs ≤ 30 minutes apart, each with LUR > 0.5). If one proceeded to resolution = 0.6 to generate five sub-CSAs (Fig. 2C), one small sub-CSA (at the northwest corner) would have population < 120,000 and no CC. Therefore, four sub-CSAs with resolution = 0.5 were retained.

To recap, among the 94 initial CSAs, 85 initial CSAs stayed intact (32 without CCs, 45 with one CC in each, and 8 with multiple CCs in each but indivisible), and 25 sub-CSAs (5 without CCs, 16 with one CC in each, and four with multiple CCs in each) were “spin-offs” from nine initial CSAs. The 85 initial CSAs and 25 sub-CSAs were derived by the same algorithm but with different resolutions and criteria in setting parameters. Together these 110 final CSAs were simply referred to as CSAs, and they varied in demographic and geographic characteristics. The mean LI was 0.83 (median = 0.86, range = 0.37–0.94) with a SD of 0.1, and the mean MSI was 0.14 (median = 0.11, range = 0.04–0.56) with a SD of 0.10. Their population size ranged from the smallest Ozona CSA of Texas (154,639) to the largest Dallas CSA (10,435,733) with mean = 2,807,040 and median = 2,309,342. Patients averagely travelled 112 minutes (median = 91 minutes, range = 57–515) to seek cancer care with a high variability (SD = 63 minutes).

**FIGURE 3:**
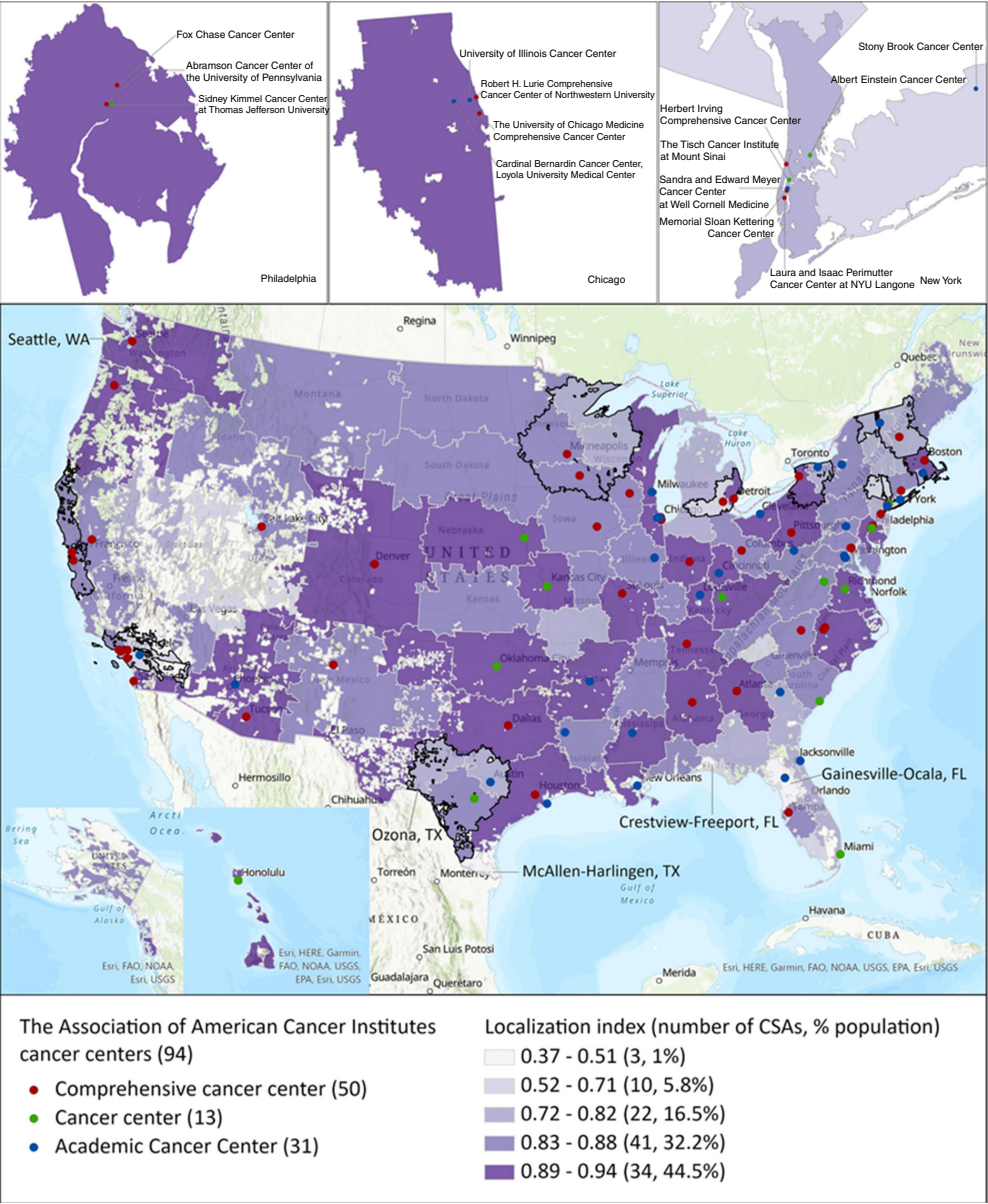
LI of 110 CSAs classified by natural breaks overlaid with 94 CCs. (Nine CSAs in black boundaries were divided into multiple sub-CSAs in gray boundaries, and the small wad at the east corner of Alaska (inset) was part of the Seattle CSA in the main map. The fragmental white patches represented large water bodies or large unpopulated land areas.)

Figure 3 depicted the spatial distribution of LI among 110 CSAs in the United States. Three CSAs in Crestview-Freeport of Florida, McAllen-Harlingen of Texas, and Gainesville-Ocala of Florida, accounting for 1% U.S. population, had the lowest LI (0.37–0.51), suggesting that most or nearly most patients traveled beyond their CSAs to seek cancer care (Fig. 1). Among the remaining 107 CSAs with LI > 0.51, the 34 CSAs with LI values in the highest category (0.89–0.94) had 44.5% population and were in large cities, such as Seattle, San Diego, Phoenix, Tucson, Denver, Oklahoma, Dallas, Houston, New Orleans, Atlanta, and Pittsburgh. The Kansas City CSA, anchored by the University of Kansas Cancer Center, had the highest LI = 0.94 and a population of 2,811,731.

In terms of presence of CCs, the 73 CSAs with at least one CC included population more than five times that in the 37 CSAs without CCs. Among these 73 CSAs, 61 CSAs had one CC, seven CSAs had two CCs in each, three CSAs in Houston, Washington DC, and Philadelphia (the left inset in Fig. 3) had three CCs in each, and the Chicago CSA with a population of 8,701,735 had four CCs and the New York City CSA with a population of 7,610,200 had six CCs (the middle and right insets in Fig. 3).

**FIGURE 4:**
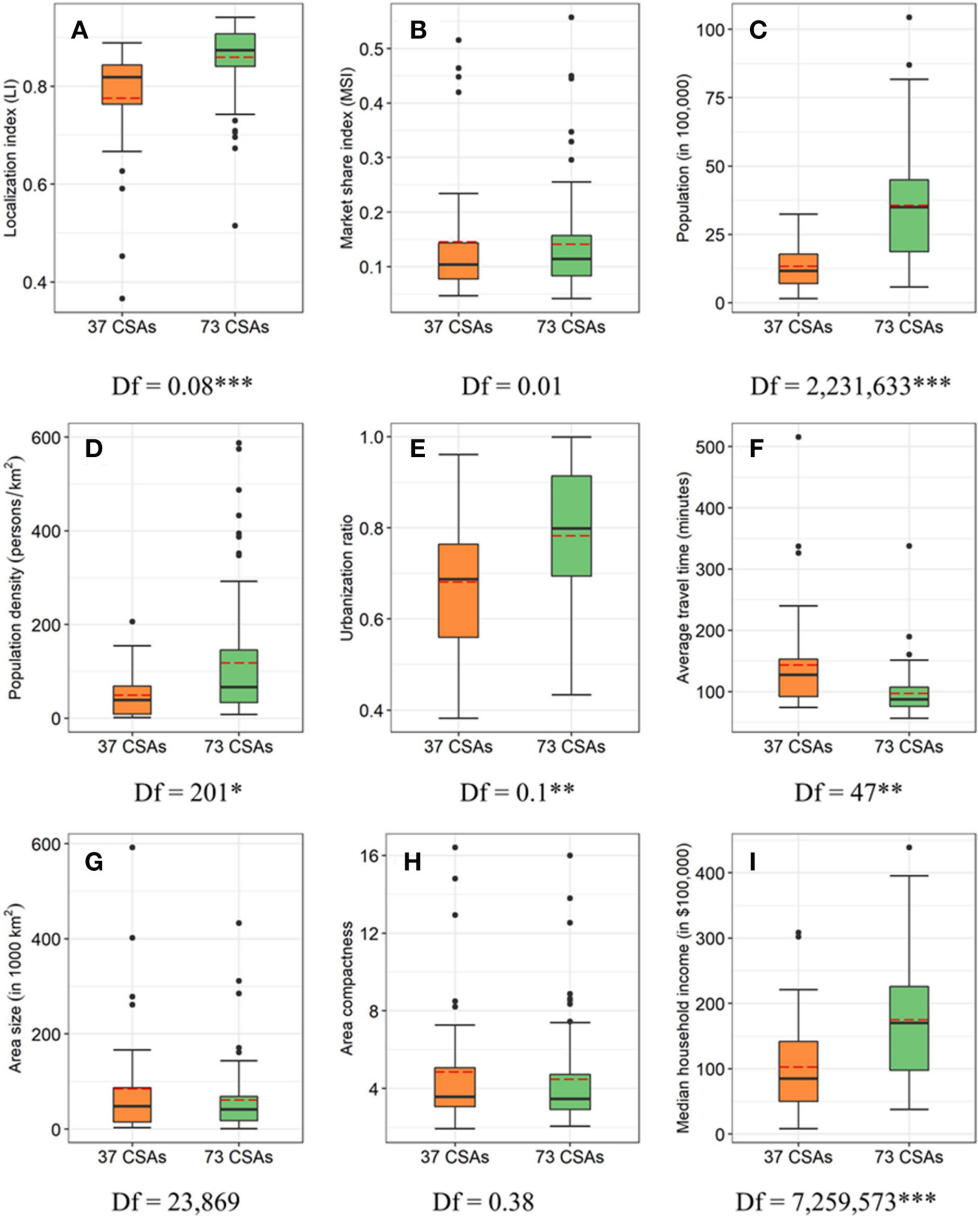
Boxplots of nine variables in across two groups: 37 CSAs without CCs and 73 CSAs with CCs. LI (**A**), MSI (**B**), population (in 100,000; **C**), population density (persons/km^2^; **D**), urbanization ratio (**E**), average travel time (minutes; **F**), area size (in 1,000 km^2^; **G**), area compactness (**H**), and median household income (in $100,000; **I**). The Df at the bottom of each boxplot referred to the difference of the mean value of the variable between two groups. ^*^, ^**^, ^***^ significant at 0.05, 0.01, 0.001. (Red dash line and black solid line in the box referred to the mean and median values of each variable across the group.)

Figure 4 plotted the characteristics of 37 CSAs without CCs and 73 CSAs with CCs. Both the mean and median values of each variable indicated the 73 CSAs with CCs had higher values in LI, population, population density, urbanization ratio, and median household income, and lower values in average travel time, area size, and compactness than the 37 CSAs without CCs. The differences in their means were statistically significant at 0.001 level for LI, population, and median household income, significant at 0.01 level for urbanization ratio and average travel time, and significant at 0.05 level for population density.

**FIGURE 5:**
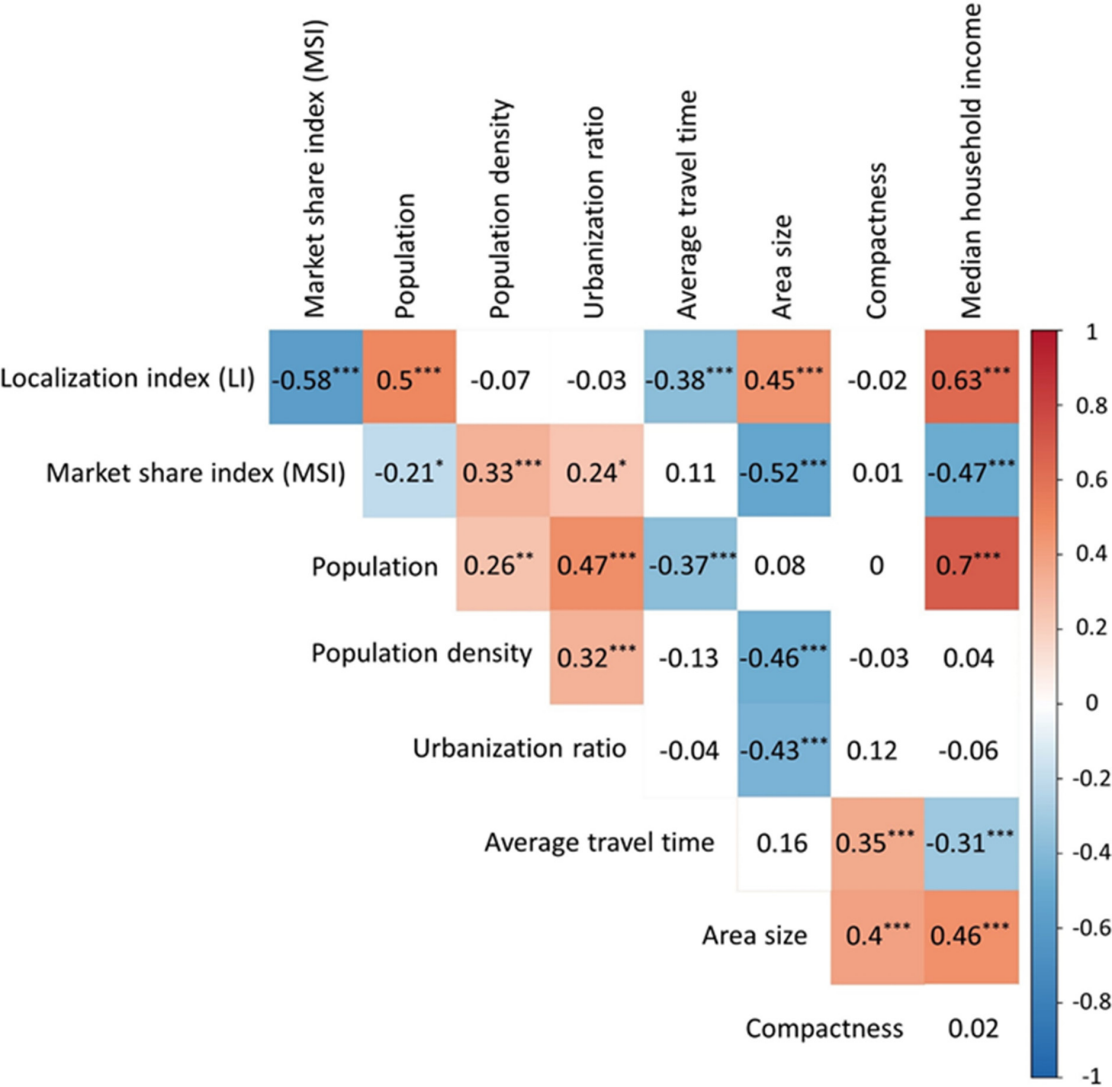
Correlation coefficient matrices of nine variables across 110 CSAs. Note that population, area size, and median household income were in logarithmic scales.

Correlation analysis on the 110 CSAs indicted that LI was positively correlated with population, area size, or median household income, especially when the latter three variables were in logarithmic scale but negatively correlated with average travel time (Fig. 5). In a multivariate regression on LI, we first eliminated median household income as it was highly correlated with other variables. The stepwise regression further eliminated other variables (population density, urbanization ratio, and compactness) that were not significant in explaining the variability of LI. The combination of population, average travel time, and area size explained 51% of the variation of LI, and all were statistically significant at 0.001 level. Their variation inflation factors indicated no significant collinearity between them.

MSI was positively correlated with population density or urbanization ratio in a linear scale but negatively correlated with population, area size, or median household income in a logarithmic scale (Fig. 5). The stepwise regression eliminated other variables (population, urbanization ratio, average travel time, and compactness) that were not statistically significant. The combination of population density, area size, and median household income, with no collinearity between them, explained 37% of the variation of MSI, and each was statistically significant (population density and area size at 0.05, and median household income at 0.001).

## Discussion

This study delineated 110 CSAs in the Unites States by a refined network community detection method while accounting for the presence of major cancer centers. The method maximized the service volumes within the units and minimized the volumes between them, and thus ensured that the CSAs reflected cancer-specific health care markets. The quality of CSAs was evidenced by high LI values with a small SD. About 99% of the U.S. population resided in CSAs for which more than 51% of utilization occurred in the same CSAs, and 76.7% of population were in CSAs above 82% of local utilization. A large majority (84%) of population were in CSAs anchored by leading cancer centers that were members of AACI. This is consistent with the purpose of cancer centers defining CAs to serve a predominance of the populations within them (2). In addition, it highlights that a significant portion (16%) of residents, mostly rural, were not readily within reach of these prominent centers.

The presence of cancer centers significantly affected the characteristics of CSAs. Patients living in CSAs with major cancer centers were most likely to utilize cancer care services provided by these cancer centers or their affiliated hospitals. These patients also experienced shorter average travel time in relatively higher density and smaller areas where cancer centers were located.

The cancer care utilization pattern captured by LI and MSI varied across CSAs. Patients living in populous CSAs were more likely to stay local for cancer care. This pattern is similar to a study by Kilaru and colleagues in which they characterized patient movement across boundaries defining health services areas and found that the large majority of urban patients sought inpatient care in the HSA in which they resided (29). Reported in the same study, more urbanized HSAs also tended to have higher LI and higher MSI (29); however, only the latter pattern was observed in this study. Larger CSAs in terms of population and areal size were associated with higher LI but lower MSI, so were the CSAs with higher median household income and shorter average travel time. One likely explanation was that such CSAs were able to support large hospitals of higher quality and better reputation and thus attracted more patients to seek cancer care within, simultaneously, the competition may create barriers to draw patients outside. Together, they (population, area, and travel time) explained more than half of the LI variation across the 110 CSAs. The combination of population density, area, and income only explained 37% of the MSI variation.

The value of the 110 derived CSAs and the scale-flexible method lies in the ability of many stakeholders to use this approach to evaluate cancer-specific care both nationally and within smaller areas. Cancer care costs in the United States are staggering and continue to rise; the national cost of cancer-related care was estimated at $185 billion in 2015 and is expected to rise to $245 billion by 2030. At the same time, notable gains in survival are being made for some cancers, due largely to new agents, such as immunotherapy (31). Given that cancer is the second leading cause of death in the United States (32), care utilization, costs, and outcomes are critical to be able to assess nationally, robustly, reproducibly, and readily. The utility of CSAs for stakeholders, such as the CMS, private insurers, health systems, cancer centers, and researchers will be high if used to understand cancer care delivery to improve efficient care and outcomes. For example, federal agencies can apply CSAs to conduct standardized comparative analyses of cancer care resources across the whole country and in different time ranges to identify regions with overuse or underuse of effective care. There are myriad examples of utilization-based service areas derived for evaluative and comparative purposes (33). For example, the Dartmouth Atlas of Healthcare derived HRRs in the United States and measured unwarranted variation (i.e., overuse or underuse not related to underlying population characteristics) in hospital-based services, costs, and outcomes (34). Similarly, in England, the National Health Service measured unwarranted variation across its service districts and sought to reduce that variation; that is—improve care delivery in regions identified as outliers (35).

CSAs will further facilitate cancer-relevant policy targeting specific regions to improve access and outcomes with affordable cost. Also, health systems and cancer centers can apply CSAs to their CAs for understanding which cancer populations are truly underlying their regional scope of cancer control and prevention efforts. Recent studies both complement this work and highlight its importance. One study examined geographic and population coverage of 102 AACI cancer centers and found that 15% of U.S. counties (∼25 million people) do not fall within an AACI cancer center's CA (36). This corresponds closely with our finding that 16% of the population lives in a CA that is not anchored by an AACI cancer center. Yet individuals in these areas with cancer will receive cancer care, including at local/regional hospitals that are not represented in the 102 AACI cancer centers. Our CSAs capture the full geographic extent and population denominator. Thus, unlike self-defined CAs, defining geographic units based on where cancer patients receive care allows for systematic and actionable comparisons and underscores the utility of the full geographic and population coverage for the United States as has been evidenced by the Dartmouth Atlas of HealthCare's HRRs.

Another recent study mapped the CAs of the 63 NCI-CCs that provide patient care nationally and found that 88% of the U.S. population resides within an NCI-CC CA (37). However, given that only about 12% of individuals with a cancer diagnosis attend an NCI-CC, the unit of “catchment area” is not the best comparative unit for care received. While CAs of NCI-CCs may serve to benchmark measure of cancer care and outcomes, they are not geographic units that capture population-based patterns of cancer care utilization (i.e., “cancer care markets”). In that recent study, cancer mortality rates were compared across the geographic regions of the 63 CAs and found a wide range of variation. While this analysis was informative, it would be even more informative if we could compare range of cancer mortality rates across 100% of cancer care delivered, not just the approximately 12% which occurs at NCI-CCs (37). This is the utility of the CSAs.

Finally, health care professionals and researchers may use the CSAs to study the geographic variation of cancer care access, utilizations, outcomes, and spending to identify effective therapies across heterogeneous populations and better care delivery models for specific populations and geographic areas. Thus, more evidence-based policies can be implemented to optimize cancer care delivery, maximize resource utilization, reduce extant disparities, and identify intervention targets. All potential users are able to use the scale-flexible method automated in a GIS tool to define CSAs in other study areas, update CSAs to meet the challenges of market changes and population movement or delineate other type of service areas (i.e., the newly defined HSAs and HRRs in very recent studies (6)) to fit their needs.

There were some limitations in this study. Medicare data were used as the basis for cancer care utilization to delineate the CSAs because it is fully population-based for individuals age 65 and older; however, nearly half of all cancer occur in younger age groups (38). Future studies will test the generalizability of CSAs to younger populations when related data are available at a population level. Also, we only included fee-for-service Medicare claims to ascertain cancer care utilization since beneficiaries enrolled in Medicare Advantage do not have claims or bills submitted for each service. To protect the privacy of patients with cancer, some Medicare data with volumes less than 11 were suppressed. Future studies may consider adding more years of data to lessen this issue. For the same reason, Medicare data were aggregated at the ZIP code level to delineate CSAs, during which several criteria including 30-minute travel time, 50% of the local utilization rate, and minimal population of 120,000 were used, these may invoke the modified area unit problem (MAUP), leading to different number of CSAs being defined. Therefore, their selection calls for consensus among health care professionals and researchers. The number of cancer centers may change with more cancer centers joining the membership of AACI. This would cause changes in the number and boundaries of CSAs. Nevertheless, AACI has rigorous membership criteria, the number of leading cancer centers may not change significantly in a few years. Also, the scale-flexible method can easily redelineate a comparable number of CSAs or allow a new number of CSAs to be set based on new major cancer centers. This study used a simple and clean set of cancer centers as a baseline to define CSAs, other satellite cancer centers or large hospitals not in the list also played important roles in cancer diagnosis and treatment (see dense flows ending at locations without cancer centers in Fig. 1). However, the providers aggregated at ZIP code level cannot be identified because of the privacy issues.

From this study, we conclude that by using the most refined network community detection method, we can delineate CSAs in a more robust, systematic, and empirical manner that incorporates existing specialized cancer referral centers. The CSAs can be used as a reliable unit for studying cancer care and informing more evidence-based cancer care policy in the United States. The cross-walk tabulation of ZIP code areas and CSAs and related programs for CSA delineation are disseminated for public access.
